# Paradoxical Immune Reconstitution Inflammatory Syndrome in SARS-CoV-2 Infection After Improvement of Chemotherapy-Induced Aplasia

**DOI:** 10.7759/cureus.46723

**Published:** 2023-10-09

**Authors:** Cristiana Canelas Mendes, Patrícia Howell Monteiro, João Madeira Lopes, António Pais de Lacerda

**Affiliations:** 1 Internal Medicine 2, Hospital Santa Maria, Centro Hospitalar Universitário Lisboa Norte, Lisboa, PRT

**Keywords:** ards, immunosuppression, chemotherapy-induced aplasia, covid-19, iris

## Abstract

Severe coronavirus disease 2019 (COVID-19) is known to manifest in two phases, with a potential worsening in the second week. The pathophysiology of the first phase is expected to be heavily influenced by viral replication while the second phase is thought to be primarily characterized by systemic inflammation. We present the case of a 42-year-old man hospitalized for severe acute respiratory syndrome coronavirus 2 (SARS-CoV-2) infection with a history of Philadelphia-positive chronic myeloid leukemia, diagnosed seven months earlier, proposed to bone marrow allotransplantation after refractory imatinib and dasatinib treatment. After an initial clinical and laboratory improvement, the patient got worse. A pulmonary CT scan showed worsening ground-glass opacities and multiple bilateral consolidations. Neutropenia was resolved, and on the same day, the patient developed progressive respiratory failure with rapidly increasing oxygen demand and distributive shock, requiring mechanical ventilation. Acute respiratory distress syndrome (ARDS) induced by paradoxical COVID-19 immune reconstitution inflammatory syndrome (IRIS) following chemotherapy-induced aplasia was equated. High-dose corticosteroid therapy was rapidly effective. IRIS occurs in patients with severe immunosuppression in response to rapid immune reconstitution and results in an uncontrolled inflammatory response to infectious agents that cause tissue damage. The inflammation associated with both IRIS and COVID-19 shares a common path in terms of immunological response. We hypothesize that in our patient, a hyperinflammation overlap exerted a synergistic effect, leading to the worsening of the disease.

## Introduction

Coronavirus disease 2019 (COVID-19), caused by severe acute respiratory syndrome coronavirus 2 (SARS-CoV-2) infection, manifests with a diverse clinical profile, ranging from asymptomatic or mild flu-like syndrome to severe viral pneumonia that may progress to acute respiratory distress syndrome (ARDS) and/or critical illness [1,2]. The symptoms of severe COVID-19 are known to manifest in two phases, with a potential worsening in the second week [1,3]. The pathophysiology of the first phase is expected to be heavily influenced by viral replication while the second phase is thought to be primarily characterized by systemic inflammation [1]. Patients with late or rapid deterioration often suffer acute respiratory failure/ARDS, acute renal injury, and multiorgan failure [2].

This article was previously presented as a meeting abstract at the 19th European Congress of Internal Medicine (ECIM 2021) from March 18 to 20, 2021 [4].

## Case presentation

A 42-year-old man was transferred to our COVID-19 ward after testing positive for SARS-COV-2. Seven months earlier, he had been diagnosed with Philadelphia-positive chronic myeloid leukemia, refractory to imatinib and dasatinib treatment. After being treated with dasatinib, a biopsy of the bone marrow showed that the patient had an accelerated phase of chronic myeloid leukemia. A bone marrow allotransplantation was suggested. In this sense, he was hospitalized 15 days before the current hospitalization to undergo conditioning therapy with fludarabine, cytarabine, and idarubicin. This medication was started six days before, for five days, uneventfully, with additional co-trimoxazole, acyclovir, and posaconazole prophylaxis. Two days before our unit admission due to intense fatigue, he started having diarrhea and had massive epistaxis, requiring a platelet transfusion. He was then transferred to the COVID-19 ward after having a positive test for SARS-CoV-2. His diarrhea has stopped, but he remained fatigued.

On physical examination, he was pale and feverish, with reduced breath sounds in the lower third of the right hemithorax. Laboratory test results showed pancytopenia with hemoglobin 10.9 g/dL (13-17.5 g/dL), leukocytes 110/µL (4000-11000/µL), platelets 17000/µL (150000-450000/µL), and elevated C-reactive protein (CRP) 2.55 mg/dL (< 0.5 mg/dl). The arterial blood gas test and chest X-ray were normal. Granulocyte colony-stimulating factor (G-CSF) for chemotherapy-induced pancytopenia was started.

Piperacillin/tazobactam was prescribed while maintaining previous prophylaxis. Methicillin-resistant Staphylococcus epidermidis was identified in blood cultures, and vancomycin was added. Microbiological culture of the tip of the central venous catheter and synchronous blood cultures were negative.

**Figure 1:**
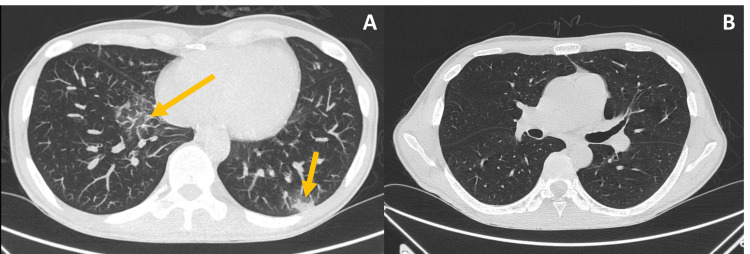
First pulmonary CT scan on day 10 after admission A - An incipient evolving inflammatory process (with arrows); B - For comparison; 1B is at the same level as the subsequent images.

Ten days after the positive SARS-CoV-2 test, and after initial clinical and laboratory improvement, the patient became worse, with fever recrudescence, worsening tiredness, dry coughing attacks, pancytopenia requiring daily transfusions of erythrocyte concentrate and platelets, and increased inflammatory parameters. A pulmonary CT scan showed small areas of parenchymal densification in the right lower lobe and irregular condensation on the edge of the left lower lobe, both of which are signs of an evolving inflammatory process (Figure 1A and Figure 1B). Facing a refractory fever in a neutropenic patient with possible pulmonary aspergillosis, antibiotics were empirically broadened to meropenem, linezolid, amikacin, amphotericin B, and caspofungin, but the galactomannan antigen was negative.

**Figure 2:**
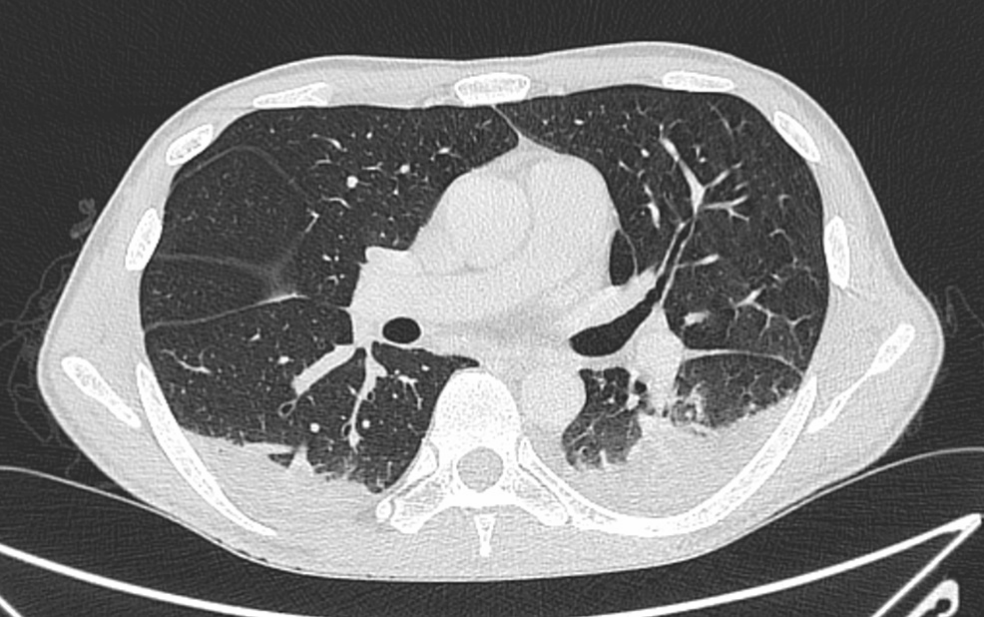
Pulmonary CT scan on day 16 after admission: SARS-COV-2 bilateral pneumonia

On the fourteenth day of hospitalization, the patient worsened, with pleuritic pain in the left hemithorax and type 1 respiratory failure requiring oxygen therapy at 2 L/min. A new pulmonary CT scan revealed bilateral consolidations of the lung parenchyma, particularly in the posterior surface of both lower lobes, suggestive of bilateral lobar pneumonia. There were some small areas of ground-glass densification in the parenchyma, namely, in the right lower lobe, middle lobe, and lingula, aspects that may reflect the extension of the inflammatory or infectious process. There was minimal pleural effusion bilaterally. Compared to the previous CT scan, a clear progression of the inflammatory/infectious process was observed. SARS-COV-2 bilateral pneumonia (Figure 2) was assumed.

The patient's condition continued to worsen, and on the sixteenth ward day, he was hypotensive, requiring aminergic support with noradrenaline (up to 150 mcg/min to maintain mean arterial pressure > 60 mmHg), tachypneic (respiratory rate of 30 cpm), severely hypoxemic, with rapidly increasing oxygen demand up to 15 L/min with a nonrebreathing mask and reduced breath sounds bilaterally in the lower thirds of the lungs.

Laboratory results showed pancytopenia: hemoglobin 7.7 g/dL, leukocytes 410/µL, platelets 11000/µL, elevated C-reactive protein 25.0 mg/dL, procalcitonin 1.62 ng/mL (< 0.5), D-dimers 1.3 µg/ml (0-0.5), ɣGT 278 U/L (0-60), total bilirubin 1.61 mg/dL (< 1.2), direct bilirubin 1.24 mg/dL (< 0.2), and ferritin level 7112 ng/mL (30-400). Severe hypoxemia (pO2 45 mmHg) was revealed on arterial blood gas test but not hyperlactatemia.

Ten hours later, laboratory results showed an increase in leukocytes to 1240/µL. He was transferred to the intensive care unit and mechanically ventilated.

Acute respiratory distress syndrome (ARDS) triggered by paradoxical COVID-19-associated immune reconstitution inflammatory syndrome (IRIS) following chemotherapy-induced aplasia was considered [4].

High-dose corticosteroid therapy (methylprednisolone 1 mg/kg/day) was started until the day after extubation (twenty-ninth ICU day) when weaning from corticosteroids at 0.5 mg/kg/day began.

**Figure 3:**
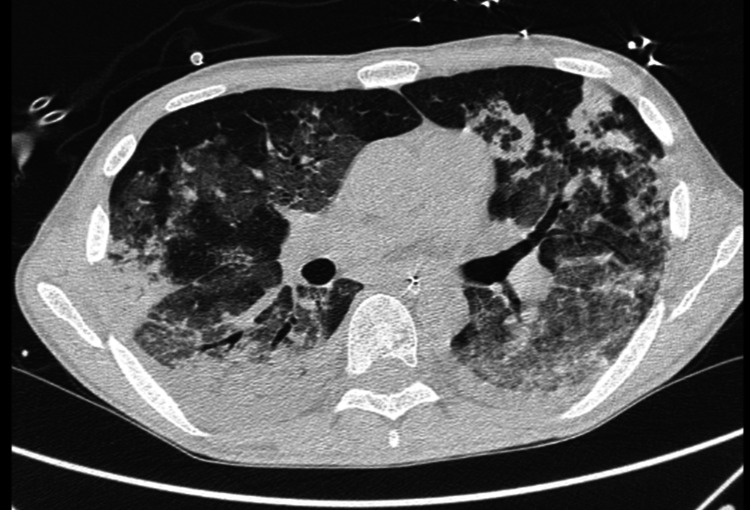
Pulmonary CT scan on day 22 after admission: SARS-COV-2 organizing pneumonia

A new pulmonary CT scan, five days later, showed a worsening of findings compatible with COVID-19-organizing pneumonia (Figure 3). A myelogram revealed progression to the blast phase of chronic myeloid leukemia and was started on ponatinib. On the twenty-eighth ICU day, he was successfully extubated and six days later, he was transferred back to our COVID-19 ward for rehabilitation. 

Following discharge, the patient had mild pulmonary sequelae detectable on a pulmonary CT scan but there was no need for supplementary oxygen therapy or medicines. The hematological illness progressed, and the patient was enrolled in a clinical trial.

## Discussion

IRIS was first described in HIV-infected patients who achieved immunological recovery after starting antiretroviral medication. However, the phenomenon is increasingly being observed in individuals who are recovering from neutropenia caused by chemotherapy [5] and represents a clinical condition of uncontrolled immune-mediated inflammation against various antigens, including pathogenic microorganisms, drugs, and unknown autoantigens, during recovery from transient immunodeficiency [6,7]. Among patients not infected with HIV, immune reconstitution can occur even if the temporary use of immunosuppressive agents is terminated [5]. Two types of IRIS have been described. In "unmasking IRIS", the immune reconstitution reveals a previously undetected infection. The term "paradoxical IRIS" refers to an exacerbation of an inflammatory illness that had already been established or was being treated. Lung consolidations typically appear in the same location as the initial lesions but as more severe manifestations of those lesions, which is a common hallmark of this condition [7]. As a result of increased tissue damage, the patient's clinical condition may deteriorate, potentially leading to death [5]. Because the level of immune suppression in non-HIV IRIS is often lower than in HIV IRIS, the immune recovery curve is also not as steep [7]. Both the radiological and clinical described features of paradoxical IRIS were evident in our patient’s presentation.

Massive production of cytokines and other proinflammatory mediators plays a key role in IRIS-associated ARDS [8].

SARS-CoV-2 infection causes a severe inflammatory response in some patients, leading to the release of large amounts of pro-inflammatory cytokines, resulting in what's known as a "cytokine storm." (CS). This is an unregulated, hyperactive immune response that results in an exaggerated inflammatory response. CS is distinguished clinically by overwhelming systemic inflammation, hyperferritinemia, hemodynamic instability, and multiorgan failure, and if it's not properly managed, it ultimately results in death [2,9].

The inflammation associated with both IRIS and COVID-19 shares a common path in terms of immunological response [8]. We hypothesize that in our patient, a hyperinflammation overlap exerted a synergistic effect, leading to the patient's clinical worsening.

In the bibliographical research that we carried out, only two similar cases were reported [3,8]. However, our patient had the most severe clinical course, presenting with ARDS and requiring mechanical ventilation. The worsening of respiratory failure and pulmonary infiltrates with a simultaneous increase in neutrophil count in only 10 hours was suggestive of a paradoxical IRIS to the already present COVID-19 in a patient who had been medicated with G-CSF for 13 days.

Corticosteroids are the most commonly employed treatment for both COVID-19 and IRIS for suppressing the hyperinflammatory response [6,7,10,11].

## Conclusions

IRIS develops in patients with severe immunosuppression as a result of fast immune reconstitution and is caused by an uncontrolled inflammatory response to infectious pathogens leading to tissue damage. This hyperinflammation associated with both IRIS and COVID-19 has similarities in terms of immune response. Judicious manipulation of host immunity and prompt recognition of IRIS are critical to limiting or avoiding its potential harm. Our hypothesis is that in our patient, the superimposition of hyperinflammation phenomena exerted a synergistic effect, leading to the deterioration of the patient's clinical status.

Treatment with corticosteroids is the most helpful and should be used at an early stage to improve the prognosis. At a time when the incidence of COVID-19 appears to be increasing again, it is important that clinicians know how to recognize this serious clinical entity that occurs in especially vulnerable patients. We consider it essential to report similar cases so that these situations can be flagged and treated energetically and appropriately.
